# IIoT-Based Remote Monitoring System for Temperature, Current, and Vibration Using PLC and Node-RED in a Data Center Cooling Compressor: A Condition-Based Maintenance Framework

**DOI:** 10.3390/s26092772

**Published:** 2026-04-29

**Authors:** Jefferson Damián Pinza Apolo, Jonathan Lizandro Bravo Robles, José Luis Dumán Zhicay, Ramiro Xavier Cazares Guerrero, Wilmer Fabian Albarracin Guarochico, Paul Francisco Baldeón Egas

**Affiliations:** Graduate School, Universidad Tecnológica Israel, Quito 170522, Ecuador; e2200367924@uisrael.edu.ec (J.L.B.R.); e2200029151@uisrael.edu.ec (J.L.D.Z.); e0401119797@uisrael.edu.ec (R.X.C.G.); walbarracin@uisrael.edu.ec (W.F.A.G.); pbaldeon@uisrael.edu.ec (P.F.B.E.)

**Keywords:** IIoT, condition-based maintenance, hybrid anomaly detection, industrial monitoring, compressors, data centers, ISO 10816-3, Node-RED

## Abstract

Climate control systems are critical to ensuring the continuous operation of data centers, as they maintain the environmental conditions required by sensitive electronic equipment. In this context, continuous supervision of refrigeration compressors is essential to prevent failures that may compromise thermal stability. This work presents the design, implementation, and experimental validation of a remote monitoring and condition-based maintenance framework built on Industrial Internet of Things (IIoT) technologies for air-conditioning compressors used in data centers. The proposed architecture integrates industrial-grade sensors for temperature, electric current, and vibration, a Siemens LOGO! programmable logic controller (PLC) for signal acquisition and scaling, a Node-RED middleware layer for data flow management, and the ThingSpeak cloud platform for remote storage and analysis. The novel contributions of this work are: (i) a fully integrated low-cost IIoT stack validated on a Copeland ZR144KCE-TF5 scroll compressor under real operating conditions over a continuous 49-day monitoring period; (ii) a hybrid anomaly detection model that combines Z-score statistical baselines with moving-average prediction error to reduce false positives from transient events; and (iii) a condition-based maintenance decision framework that maps the three monitored variables to ISO 10816-3 vibration severity zones and manufacturer-referenced thermal and electrical thresholds, producing recommended maintenance actions. The framework was applied to the acquired dataset, confirming predominantly stable operation (93.4% of samples in ISO 10816-3 Zones A–B) while detecting an emergent mechanical-wear trend (5.64% of samples in Zone C) concentrated in the final days of the monitoring period and demonstrating the feasibility of the proposed architecture as a scalable and replicable solution for condition monitoring and maintenance decision support in critical technological infrastructures.

## 1. Introduction

The reliable operation of data centers largely depends on the stability of their support systems, among which climate control plays a fundamental role. The IT equipment installed in these infrastructures generates a considerable thermal load during operation, which makes it necessary to implement cooling systems capable of maintaining controlled environmental conditions. Several studies have identified thermal management as a critical factor for ensuring the continuous operation of data centers and for preventing failures associated with equipment overheating [1,2,3].

Within these systems, compressors are among the most important components, as they drive the refrigeration process that dissipates the heat generated by the technological equipment. A failure in this type of device can lead to temperature increases that compromise the availability of IT services hosted in the data center. For this reason, continuous monitoring of compressor operating variables has become a key element of modern industrial maintenance strategies [4,5,6].

In recent years, the development of Industrial Internet of Things (IIoT) technologies has opened new possibilities for the remote monitoring of industrial assets. These technologies enable the integration of sensors, control systems, and data analysis platforms to obtain real-time information on equipment operating status. The adoption of IIoT architectures has significantly improved the monitoring capabilities of complex industrial systems [7] and has enabled more effective diagnostics through data-driven approaches [8]. Recent surveys have further documented the expansion of these capabilities toward predictive maintenance and intelligent asset management [9].

In this context, continuous monitoring of variables such as temperature, vibration, and power consumption can provide relevant information about compressor behavior and enable the detection of anomalous conditions that may anticipate potential failures. This approach is part of predictive maintenance strategies, in which the analysis of sensor data makes it possible to identify behavioral patterns associated with potential failures before they occur [10]. Systematic reviews of machine learning methods applied to this domain have confirmed the growing relevance of data-driven approaches for industrial equipment [5], including applications involving digital twin frameworks for health prognostics [11].

In parallel, the development of data integration platforms has facilitated the connection between industrial automation systems and cloud-based analytics services. Flow-based programming tools such as Node-RED allow the integration of industrial devices and web services through flexible communication architectures [12,13]. These tools have been applied in edge-to-cloud IIoT scenarios for remote monitoring and industrial data analysis [14,15,16].

Based on this technological context, this work proposes the design, implementation, and experimental validation of a remote monitoring and condition-based maintenance framework based on Industrial Internet of Things technologies for air-conditioning compressors used in data centers. The developed system integrates industrial sensors for measuring temperature, electric current, and vibration; a programmable logic controller (PLC) for data acquisition; the Node-RED platform for managing information flows; and the ThingSpeak platform for remote data storage and visualization. The proposed architecture makes it possible to record and analyze the operational behavior of the compressor, providing a support tool for continuous monitoring and for the implementation of condition-based maintenance strategies in critical technological infrastructures.

While individual components such as PLC-based acquisition, Node-RED middleware, and cloud IoT platforms are well documented in the literature, most existing studies focus on isolated aspects of IIoT integration or rely on controlled laboratory datasets. In contrast, this work presents an integrated, field-validated framework with four distinct scientific contributions. The first contribution is a replicable low-cost IIoT architecture combining a Siemens LOGO! PLC, Node-RED middleware, and the ThingSpeak cloud platform, experimentally validated on a Copeland ZR144KCE-TF5 scroll compressor operating in a real data center environment; unlike prior implementation reports, the system was evaluated over a continuous 49-day monitoring period under actual industrial conditions, providing empirical evidence of long-term operational stability. The second contribution is a hybrid anomaly detection model that combines a Z-score statistical baseline with a moving-average prediction-error criterion, an approach that reduces false positives associated with transient startup events, which is a known limitation of purely static threshold methods [17]; a direct quantitative comparison against the static Z-score baseline is reported in this manuscript. The third contribution is a condition-based maintenance decision framework that maps the three monitored variables (temperature, current, and vibration) to ISO 10816-3 [18] vibration severity zones and manufacturer-referenced thermal and electrical thresholds, producing explicit maintenance actions for six operating conditions and validated by applying it to the recorded 49-day dataset. The fourth contribution is a critical assessment of the scope and limitations of the proposed framework, explicitly distinguishing between condition monitoring (fully validated in this work) and predictive maintenance (identified as a direction for future work requiring labeled fault datasets); this positioning is intended to support reproducibility and realistic expectations in similar IIoT deployments.

The remainder of this paper is organized as follows. Section 2 describes the materials, sensors, and system architecture. Section 3 presents the experimental results, the statistical analysis, the hybrid anomaly detection model, and its comparison against the static Z-score baseline. Section 4 discusses the results, introduces the validated condition-based maintenance framework, and compares the proposed architecture with existing commercial solutions. Section 5 summarizes the conclusions and future work.

## 2. Materials and Methods

This research follows an applied and experimental approach focused on the design and implementation of a monitoring system for the operating variables of an industrial compressor using Industrial Internet of Things (IIoT) technologies. This approach enables the integration of sensors, control systems, and data analysis platforms to supervise the operating behavior of industrial equipment [7]. Edge and cloud integration frameworks have demonstrated the effectiveness of this paradigm for enabling data-driven decision-making in industrial cyber-physical systems [19,20].

The methodology is structured in three main phases: (i) design of the data acquisition system, (ii) implementation of the monitoring system, and (iii) recording and analysis of the collected data. Each stage progressively develops the technological architecture required for the acquisition, transmission, and analysis of the variables associated with compressor operation.

### 2.1. Selection of Sensors and Instrumentation

To ensure accurate acquisition of condition monitoring data and to guarantee the replicability of the proposed IIoT architecture, industrial-grade sensors were selected based on the operating limits of the Copeland ZR144KCE-TF5 scroll compressor used in this study [21,22].

To avoid information loss due to sensor saturation during transient states or severe mechanical failures, the measurement ranges were defined above the maximum nominal values specified by the compressor manufacturer [23].

All selected sensors use a standard 4–20 mA analog current loop, which offers high immunity to the electromagnetic noise typical of industrial environments. This transmission method allows direct and reliable integration with the analog input modules of the PLC-based acquisition system [24].

The measurement ranges for each variable were established considering the operating characteristics of the compressor:

- Current: A measurement range of 0–100 A was selected because the compressor’s maximum continuous current (MCC) is 61.5 A, which allows anomalous conditions to be captured without saturating the sensor.
- Temperature: A range of 0–200 °C was selected to safely monitor the compressor discharge line, which can exceed 100 °C under abnormal operating conditions.
- Vibration: A range of 0–50 mm/s RMS was selected to capture both the baseline vibration level of the compressor (approximately 15 mm/s RMS, converted from the manufacturer’s specification of 4.5 mils peak-to-peak) and possible increases associated with mechanical wear.

Table 1 presents the technical specifications of the instrumentation used in the monitoring system.

### 2.2. Data Acquisition System Design

In the first phase, the design of the data acquisition system responsible for capturing the operational variables of the compressor under study was carried out. For this purpose, temperature, electrical current, and vibration were defined as monitoring variables, parameters commonly used in condition monitoring systems for industrial machinery [25].

**Figure 1:**
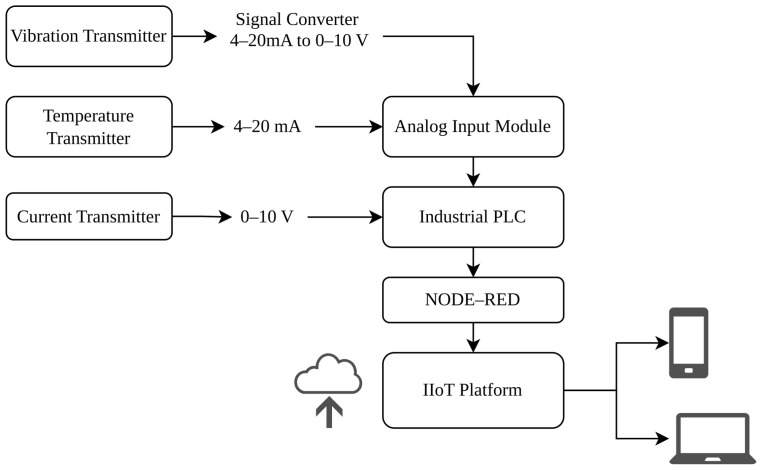
Block diagram of the IIoT-based monitoring system for supervising operational variables of the industrial compressor. Arrows indicate the direction of signal flow from sensors to the IIoT platform. The signal converter transforms the 4–20 mA output of the vibration transmitter into a 0–10 V signal compatible with the analog input module.

The acquisition system was structured through an instrumentation panel that integrates the corresponding sensors and the necessary elements for collecting the signals generated during the operation of the compressor. As illustrated in Figure 1, this design makes it possible to obtain real-time information about the operational state of the equipment, which constitutes a fundamental element for the implementation of data-driven monitoring strategies [26].

### 2.3. Implementation of the Monitoring System

**Figure 2:**
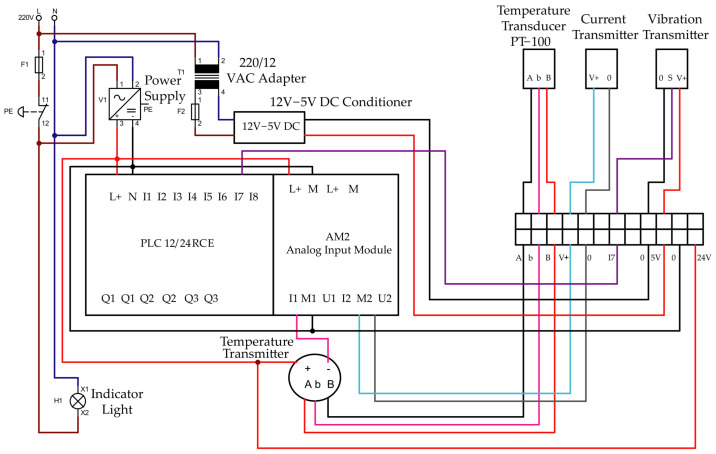
Electrical diagram of the monitoring system. Brown lines indicate the AC Line (L) conductor; blue lines indicate the AC Neutral (N); red lines indicate the DC positive supply (+); black lines indicate the DC negative or ground (GND) connections; light blue, purple, and pink lines indicate the signal lines of the different sensors.

The second phase of the methodological process consisted of implementing the monitoring system responsible for processing and transmitting the data generated by the sensors. For this stage, the Node-RED visual programming platform (version 4.1.0) was used, which allows the integration of industrial devices, communication protocols, and analysis services through configurable data processing flows [12,15,27].The electrical wiring configuration of the monitoring system is presented in Figure 2.

Node-RED functions as an intermediate data processing and integration layer within the IIoT architecture. It is responsible for receiving data from the PLC via Ethernet-based communication, structuring the information, and transmitting it to the cloud platform [27].

The data processing flow in Node-RED includes data formatting, validation, and preparation for transmission using HTTP-based communication with the ThingSpeak API. This modular approach allows flexible integration with industrial devices and cloud services, enabling scalable monitoring solutions.

Within the PLC, signal scaling routines were implemented to convert the analog outputs of the sensors into the corresponding engineering units:

- Temperature: 0–200 °C
- Current: 0–100 A
- Vibration: 0–50 mm/s RMS

### 2.4. Signal Processing in the PLC

The signals generated by the industrial sensors used in the monitoring system are transmitted through a standard 4–20 mA current loop, widely used in industrial applications due to its high immunity to electromagnetic noise and its ability to reliably transmit information in industrial environments [28].

Within the PLC, these analog signals are acquired through the analog input module, where they are converted into digital values by the controller’s internal analog-to-digital converter (ADC). This process allows the physical signals coming from the sensors to be transformed into digital data that can be processed by the control system [29].

Subsequently, a range validation process is implemented to verify that the acquired values are within the established operating limits for each monitored variable. If the signal falls outside the expected range, the system discards the reading and performs a new data acquisition. This verification makes it possible to detect potential signal anomalies, such as sensor failures or out-of-range conditions [24].

Once the signal has been validated, linear scaling routines are applied to convert the obtained digital values into engineering units corresponding to each monitored physical variable. This process is performed through gain and offset operations, allowing the digital signal to be transformed into interpretable physical magnitudes such as temperature, electric current, or mechanical vibration. This conversion process allows the data to be interpreted in terms of the monitored physical variables, facilitating their subsequent analysis within the monitoring system [30].

Subsequently, each validated reading is temporarily stored as a sample within the acquisition cycle. The system continues performing successive readings until a predefined number of samples is reached, which helps reduce the effect of noise or momentary fluctuations in the signal. Once this number of samples is reached, the PLC calculates the average value of the acquired readings, thus obtaining a more stable representation of the state of the monitored variable.

During system operation, the monitored variables are periodically acquired through the PLC scan cycle, allowing continuous recording of the operational behavior of the compressor. The processed data are subsequently transmitted to the Node-RED-based integration platform for processing and transmission to the cloud monitoring infrastructure [31]. Finally, the system waits for the defined transmission time before restarting the acquisition cycle, thus allowing continuous operation of the monitoring process.

It is important to note that the signal processing approach implemented in this study is based on time-domain averaging, which provides a simple and robust method for noise reduction in industrial environments. However, more advanced techniques such as digital filtering, frequency-domain analysis, or spectral decomposition were not implemented in the current system.

These advanced methods could significantly enhance the diagnostic capabilities of the monitoring system, particularly for vibration analysis, and are proposed as future work.

**Figure 3:**
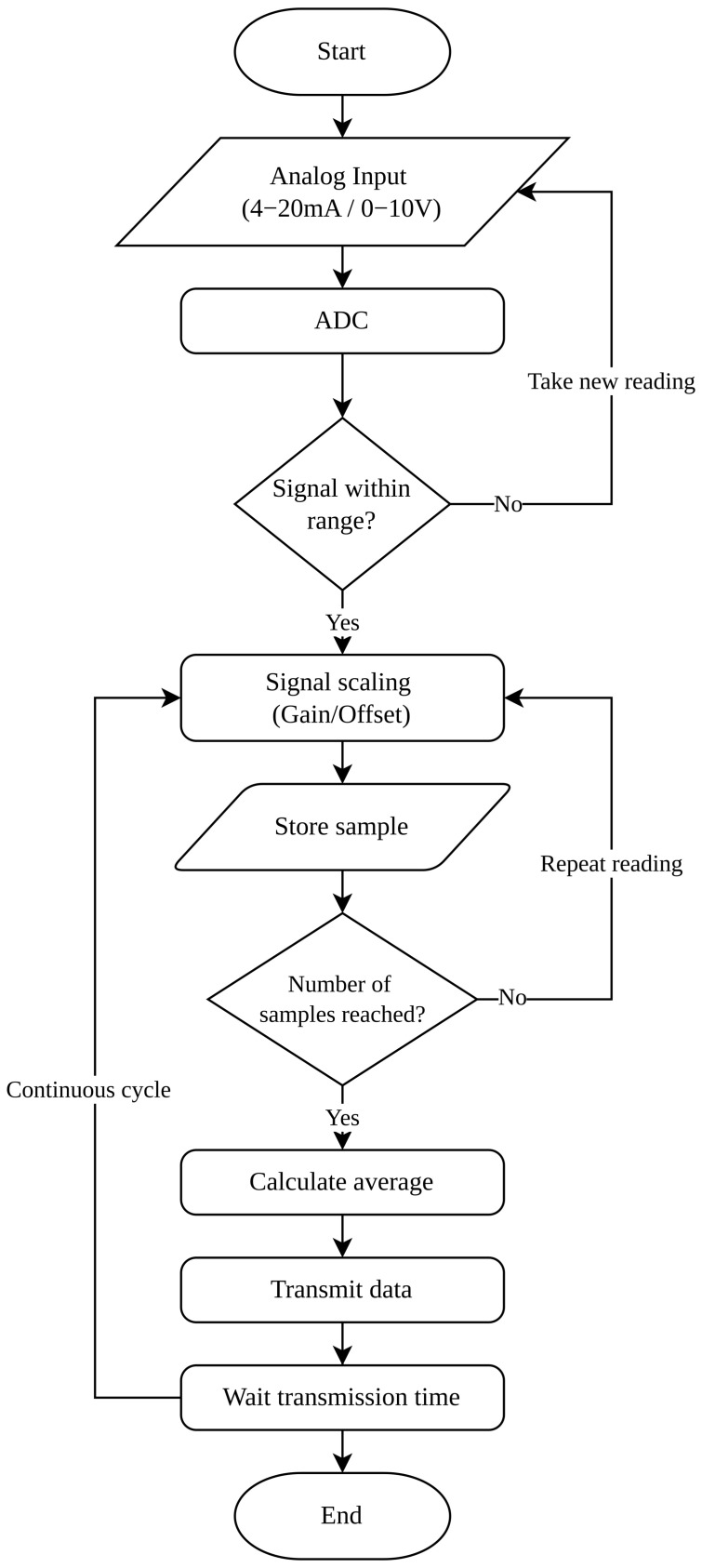
Analog signal processing flow within the PLC.

Figure 3 shows the signal processing flow implemented within the PLC for the acquisition, validation, and conversion of analog signals coming from the industrial sensors.

For the implementation of these signal processing routines, the Siemens LOGO! Soft Comfort programming environment (version 8.4.1) was used, which allows the development of control logic through functional blocks designed for the processing of analog signals from industrial sensors.

Within the implemented program, scaling, comparison, and signal conditioning blocks are used to convert the analog signals acquired by the PLC into usable values within the monitoring system. These blocks allow the measured values to be adjusted to the defined ranges for each variable, ensuring the correct interpretation of the data obtained during system operation.

**Figure 4:**
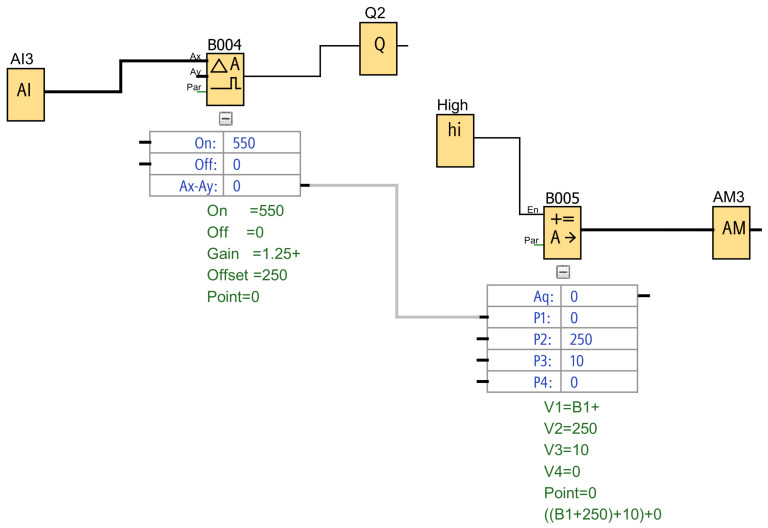
Implementation of analog signal processing in the Siemens LOGO PLC using the LOGO! Soft Comfort programming environment.

Figure 4 shows an example of the implementation of the signal processing logic within the PLC using the LOGO! Soft Comfort programming environment.

The control logic was implemented using the Function Block Diagram (FBD) programming language, one of the five standard IEC 61131-3 [32] languages, which provides an intuitive graphical representation suitable for analog signal processing applications [33].

### 2.5. Operational Flow of the Monitoring System

The developed monitoring system follows a distributed architecture in which field devices, data processing systems, and cloud storage platforms interact to enable remote supervision of industrial equipment [34].

In the first stage of the process, the sensors installed in the compressor generate analog signals proportional to the monitored physical variables, such as temperature, vibration, and electric current. These signals are transmitted to the PLC-based data acquisition system through the industrial 4–20 mA current loop [14].

Subsequently, the PLC acquires these signals through its analog input modules, where analog-to-digital conversion and initial data processing are performed using scaling and range validation routines. This processing stage enables the conversion of the acquired measurements into engineering units corresponding to each physical variable [19].

Once processed, the data are transmitted to the Node-RED integration platform, which acts as an intermediary between the industrial data acquisition system and the cloud storage services. Within Node-RED, processing flows are implemented to structure the data and prepare it for transmission using communication protocols based on web services [35].

Finally, the data are sent to the ThingSpeak monitoring platform through its application programming interface (API), where they are stored and visualized using time-based graphs that allow analysis of the compressor’s operational behavior over time [36].

**Figure 5:**
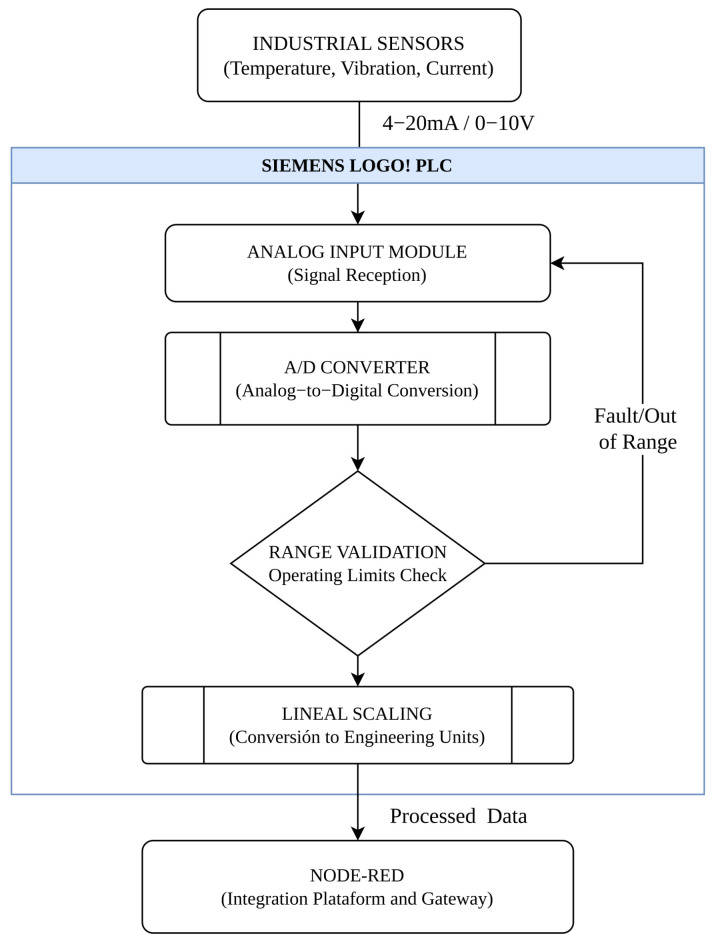
Operational flow of the monitoring system describing data acquisition in the PLC, processing in Node-RED, and transmission to the ThingSpeak IIoT platform for storage and visualization.

Figure 5 shows the general operational flow of the implemented monitoring system, from signal acquisition at the sensors to data storage and visualization on the cloud monitoring platform.

### 2.6. Implementation of the PLC-Based Monitoring Panel

For the acquisition and management of operational variables, a monitoring panel based on a programmable logic controller (PLC) was implemented. These devices are widely used in industrial applications due to their robustness, reliability, and ease of integration with field devices [33]. PLCs enable data acquisition, signal processing, and communication with external systems, making them a widely adopted solution in industrial automation and supervision systems.

In this work, a Siemens LOGO! 12/24RCE PLC (Siemens AG, Munich, Germany) was used, which provides analog inputs with a range of 0–10 V that allow the connection of industrial transducers for the measurement of physical variables. These inputs enable the integration of sensors operating with standardized analog signals, facilitating the acquisition of variables such as pressure, temperature, or electric current within the monitoring system [37].

**Figure 6:**
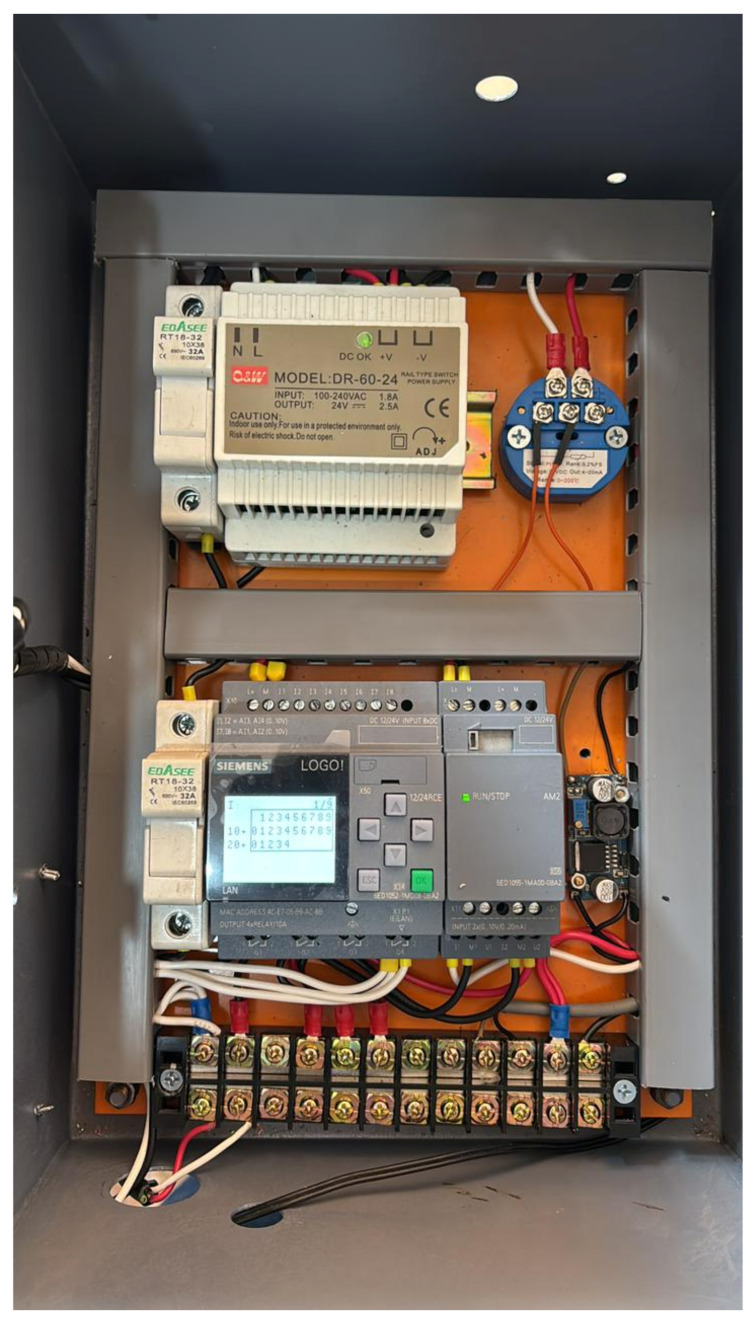
Monitoring panel implemented using a Siemens LOGO PLC.

The monitoring panel includes a 24 VDC switching power supply (DR-60-24, 60 W, 220 V AC/24 V DC; C&S Electric, Shanghai, China), electrical protection elements including fuses and fuse holders (Shanghai Ebasee Electric Co., Ltd., Shanghai, China), a signal conditioning and analog output expansion module (LOGO! AM2 AQ; Siemens AG, Munich, Germany), and terminal blocks for connecting the sensors installed on the compressor. The protection elements safeguard the equipment against overcurrents or electrical faults, while the terminal blocks facilitate the organized connection of the different field devices. This configuration allows the acquisition of variables to be centralized within a single control system, as shown in Figure 6 [29].

Additionally, the PLC executes the signal acquisition and processing logic described in the monitoring system, enabling tasks such as validation, scaling, and temporary storage of data prior to transmission. This architecture facilitates the integration of the monitoring panel with supervisory platforms and remote monitoring systems, contributing to improved availability of information regarding the operational state of the compressor.

### 2.7. IIoT Communication Architecture

The integration of industrial monitoring systems within IIoT architectures requires communication between field devices, data processing systems, and cloud storage platforms.

**Figure 7:**
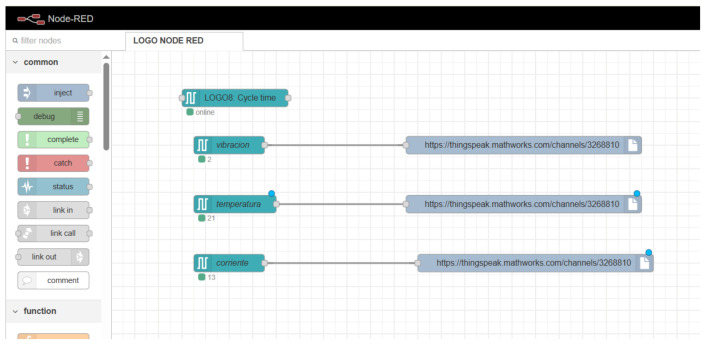
Node-RED visual programming interface used for IIoT integration. Teal-colored nodes represent LOGO8 PLC input variables (vibration, temperature, and current); gray nodes represent HTTP output connections to the ThingSpeak cloud platform (https://thingspeak.mathworks.com/channels/3268810, accessed on 23 April 2026); blue dots indicate active wire connections between nodes; green indicators with numeric labels show the message count processed by each node.

In the proposed architecture, the PLC acquires the signals from the sensors installed in the compressor and transmits the data to the Node-RED platform, where the initial processing of the information is performed. Figure 7 illustrates the Node-RED flows configured for each monitored variable.

Subsequently, Node-RED sends the data to the cloud monitoring platform, enabling its storage and remote visualization. This type of architecture facilitates interoperability between different devices and systems within connected industrial environments [2,36].

### 2.8. Communication Protocol and System Configuration

The communication between the PLC and the Node-RED platform was established through an Ethernet-based TCP/IP network, enabling reliable and continuous data transmission within the industrial environment. The PLC operates as the data acquisition unit, periodically sending processed sensor data to the Node-RED platform for further handling and cloud integration.

The Node-RED platform was deployed on an Acer Nitro 5 (AN515-57) laptop computer with the following specifications: 11th Gen Intel Core i5-11400H processor at 2.70 GHz, 32 GB RAM, running Windows 11 Home. This configuration ensures sufficient computational capacity for real-time data processing and communication management within the IIoT architecture.

The data transmission interval was configured at 1 s, taking advantage of a Student License of the ThingSpeak platform, which provides a significantly higher message capacity compared to the standard free tier. Unlike the free version, which imposes a minimum update interval of 15 s per channel, the licensed configuration used in this study supports high-frequency data acquisition without rate-limiting constraints. This configuration enables a temporal resolution adequate for capturing transient behaviors such as compressor startup events and load fluctuations. For the condition monitoring application described in this work, a 1-s resolution is considered sufficient to characterize the dynamic behavior of the monitored variables, given that the thermal, electrical, and mechanical phenomena associated with compressor operation evolve over timescales of seconds to minutes rather than milliseconds.

The communication between Node-RED and the ThingSpeak cloud platform was implemented using HTTP requests through the ThingSpeak REST API, ensuring interoperability and ease of integration with web-based services.

Although the system operates under a continuous data transmission scheme, factors such as network latency and cloud processing time may introduce minor delays. However, these delays are not critical for condition monitoring applications, where second-level resolution is generally sufficient for capturing system dynamics.

### 2.9. Cloud Monitoring and Data Logging

**Figure 8:**
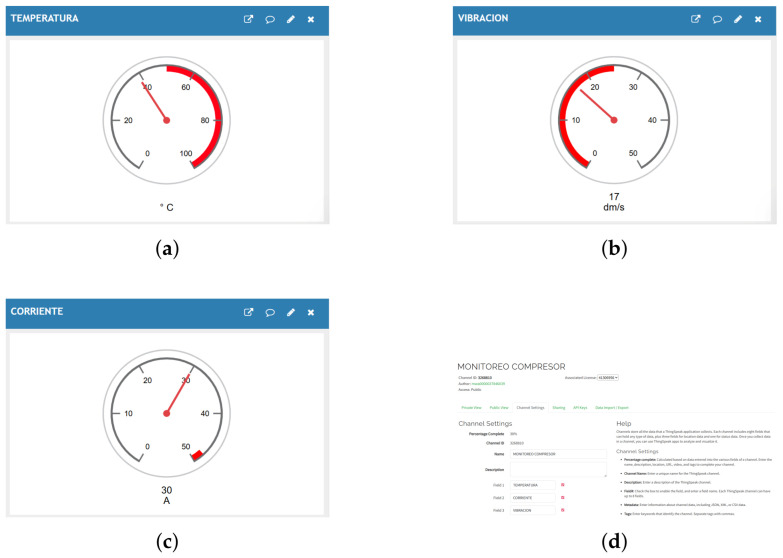
Visualization of the monitored variables on the ThingSpeak platform. (**a**) Compressor temperature. (**b**) System vibration. (**c**) Compressor electric current. (**d**) Configuration of the monitoring channel on the IIoT platform.

For the storage and visualization of the collected data, the ThingSpeak platform was used, which allows information from connected devices to be recorded and represented through time-based graphs, as illustrated in Figure 8d.

Within the implemented architecture, Node-RED establishes communication with ThingSpeak through its API, enabling the automatic transmission of the monitored variables—temperature, vibration, and electric current (Figure 8a–c)—to a previously configured channel on the platform.

Continuous data storage allows the generation of compressor operational histories, which can be used to identify trends and detect anomalies [4]. This capability supports the development of predictive maintenance strategies in cloud-connected IIoT environments [38,39].

### 2.10. Cybersecurity Considerations

Given that the proposed system relies on cloud-based data transmission and remote monitoring, basic cybersecurity measures were considered. Communication between Node-RED and the ThingSpeak platform was implemented using HTTP requests through the platform’s API.

Although encryption and advanced authentication mechanisms were not implemented in this study, API keys were used to control access to the communication channel. Future improvements should include secure communication protocols (HTTPS), authentication layers, and data encryption to ensure the integrity and confidentiality of transmitted information in industrial environments.

### 2.11. Limitations of the Study

A quantitative evaluation of system-level performance metrics such as communication latency, packet loss rate, and sensor measurement accuracy was not conducted in this study. Although the implemented architecture enables real-time data acquisition and transmission, the absence of these metrics limits the ability to fully assess system performance under different network conditions and industrial environments.

Regarding the anomaly detection model, standard classification metrics such as precision, recall, and F1-score were not computed, as the dataset corresponds exclusively to normal operating conditions without labeled fault events. The absence of ground-truth fault labels is a recognized limitation in condition monitoring studies conducted on operational industrial equipment, where fault injection or controlled degradation experiments are not always feasible [5,10].

In this context, the reported anomaly counts serve as an indicative measure of system sensitivity rather than validated detection performance. Future work will focus on: (i) conducting fault injection experiments to generate labeled datasets; (ii) computing precision, recall, and F1-score against verified fault conditions; (iii) evaluating latency and network reliability under varying load conditions; and (iv) performing sensor calibration against reference instruments to quantify measurement uncertainty.

Additionally, sensor accuracy was not validated against calibrated reference instruments, which may introduce measurement uncertainty in the recorded variables. The generalizability of the proposed architecture beyond the single compressor unit evaluated in this study also remains to be assessed in multi-unit deployments.

## 3. Results

This section presents the experimental results obtained from the monitoring system implemented for the industrial compressor. The monitored variables include temperature, electric current, and vibration, which are commonly used parameters for evaluating the operational condition of rotating machinery [24].

Once the monitoring and data acquisition system was implemented, the collected information from temperature, electric current, and vibration measurements was analyzed in order to evaluate the operational behavior of the monitored equipment. The continuous recording of these variables constitutes a fundamental element in industrial supervision systems, as it allows the identification of variations in operating conditions and the detection of potential anomalies in equipment performance [4].

For data processing and visualization, the analysis tools integrated into the ThingSpeak platform were used. This platform enables the representation of recorded data through time-based graphs and allows continuous monitoring of the behavior of the observed variables. In addition, ThingSpeak facilitates data analysis in Industrial Internet of Things (IIoT) environments by providing cloud storage capabilities, data visualization, and remote access to records generated by connected devices.

The analysis of the collected data was carried out by considering the temporal evolution of each variable, which allowed the identification of maximum and minimum values as well as potential critical operating points. This type of analysis is particularly relevant in machinery condition monitoring applications, as it enables the detection of deviations from normal operating values and supports the evaluation of the operational state of the system [10].

Additionally, MATLAB tools integrated within the ThingSpeak platform were used for data analysis (version 25.2.0.3150157, R2025b Update 4). The statistical methods applied, including descriptive analysis, Z-score-based outlier detection and correlation assessment follow well-established procedures for time-series condition monitoring data [10,17].

The implemented routines focused on extracting descriptive statistical parameters, including maximum, minimum, and average values, providing an initial assessment of the compressor’s operational behavior. Although advanced signal processing techniques were not implemented at this stage, this approach allows the establishment of baseline references for future analysis.

The obtained results demonstrate that the integration of industrial sensors, data acquisition systems, and cloud-based analysis platforms enables the development of effective solutions for remote monitoring of operational variables. This type of IIoT-based architecture facilitates continuous data collection and analysis, contributing to the development of predictive maintenance strategies and intelligent management of industrial assets [7,14].

### 3.1. Temperature Monitoring Results

Temperature is one of the most relevant variables for evaluating the operational condition of industrial equipment, since abnormal increases may indicate overload conditions, lubrication problems, or incipient failures in system components. For this reason, continuous temperature monitoring is widely used in condition-based maintenance strategies and machinery diagnostics [10].

**Figure 9:**
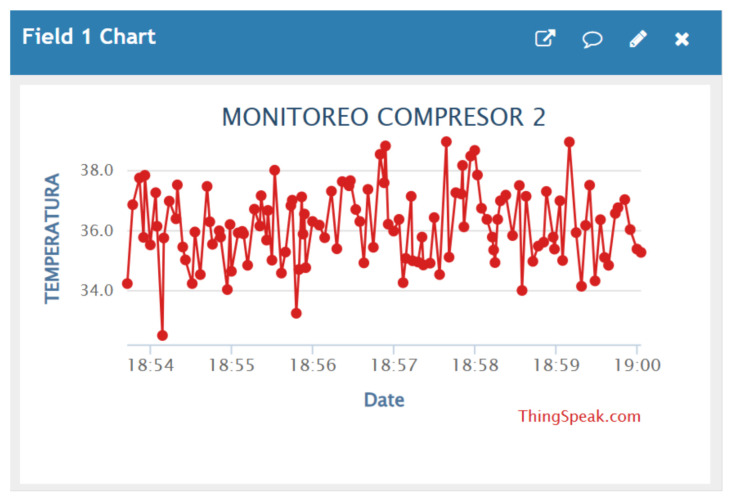
Monitoring of compressor temperature obtained through the ThingSpeak platform (available at https://thingspeak.mathworks.com, accessed on 23 April 2026).

In the present study, temperature data corresponding to the operation of the compressor were recorded using the implemented IIoT-based monitoring system. The measured values were stored and represented through time-based graphs, allowing the evolution of the variable to be observed during the analyzed operating period [6], as illustrated in Figure 9.

From the recorded temperature data, the characteristic values shown in Table 2 were obtained.

**Figure 10:**
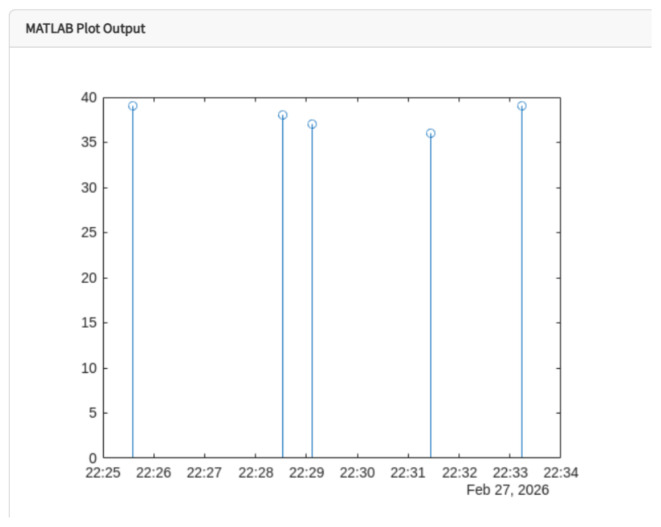
Discrete visualization of compressor temperature obtained through MATLAB processing.

For the discrete analysis of the recorded data, MATLAB routines were implemented in order to visualize the temperature signal and identify the maximum values reached during compressor operation, as shown in Figure 10.

### 3.2. Electric Current Monitoring Results

Electric current is one of the most important variables for evaluating the operational condition of equipment driven by electric motors, such as industrial compressors. Significant variations in current levels may indicate overload conditions, mechanical failures, or deviations from normal operating conditions [28].

**Figure 11:**
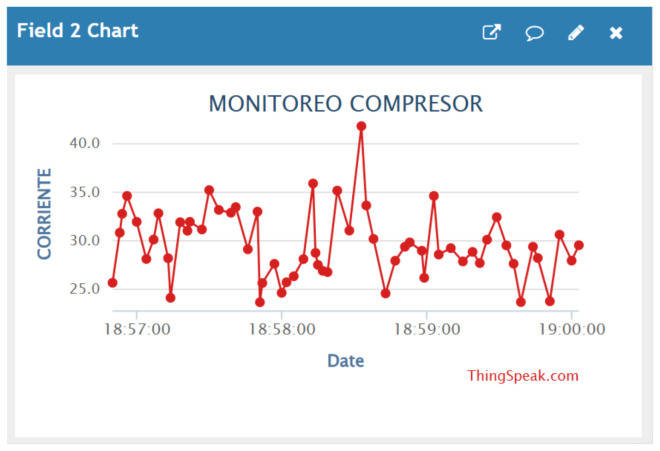
Monitoring of compressor electric current obtained through the ThingSpeak platform (available at https://thingspeak.mathworks.com, accessed on 23 April 2026).

Electric current data corresponding to the compressor operation were recorded using the implemented IIoT monitoring system. The measured values were stored and represented through time-based graphs, allowing the electrical behavior of the compressor to be observed during the monitoring period [40], as depicted in Figure 11.

The main electric current values obtained during the monitoring process are summarized in Table 3.

**Figure 12:**
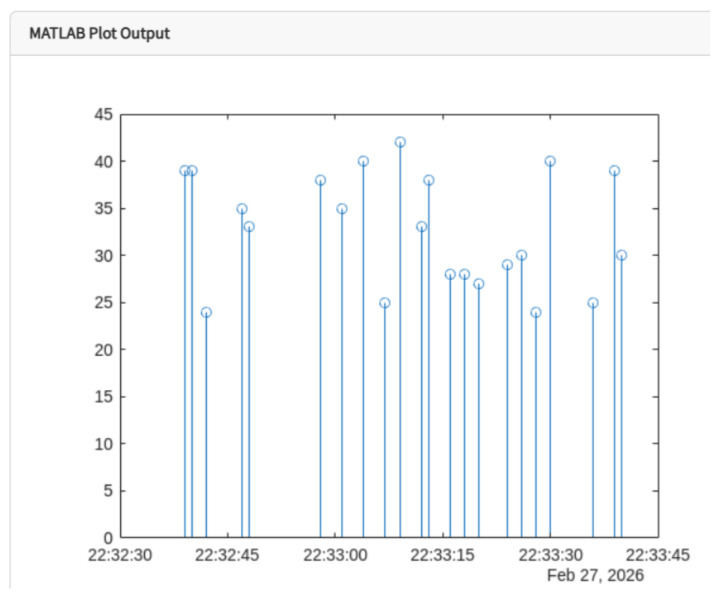
Discrete visualization of the compressor electric current using MATLAB.

For the discrete analysis of the electric current signal, MATLAB processing routines were implemented to visualize and analyze the current behavior during compressor operation, as shown in Figure 12.

### 3.3. Vibration Monitoring Results

Vibration analysis is one of the most widely used techniques in the condition monitoring of rotating machinery, as it enables the early detection of mechanical faults such as imbalance, misalignment, bearing wear, or structural issues in industrial equipment [22].

**Figure 13:**
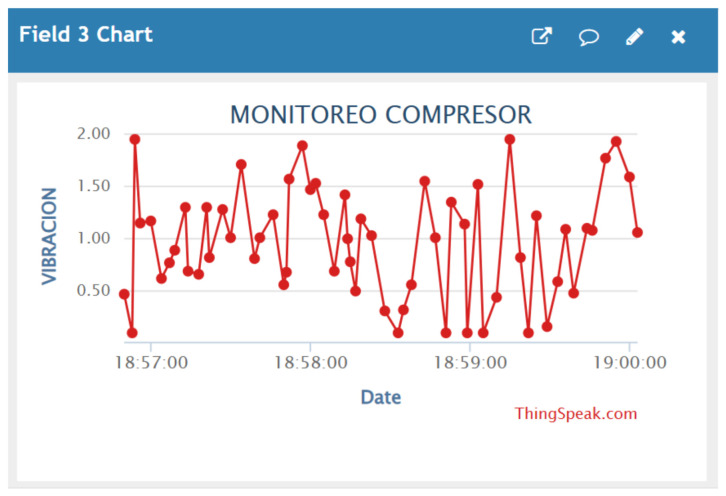
Monitoring of compressor vibration obtained through the ThingSpeak platform (available at https://thingspeak.mathworks.com, accessed on 23 April 2026).

In the present study, vibration data corresponding to compressor operation were recorded using the implemented monitoring system. The measured values were stored and represented through time-based graphs, allowing the dynamic behavior of the vibration signal to be observed during the monitoring period [41], as illustrated in Figure 13.

The main vibration values obtained from the monitoring system are summarized in Table 4.

**Figure 14:**
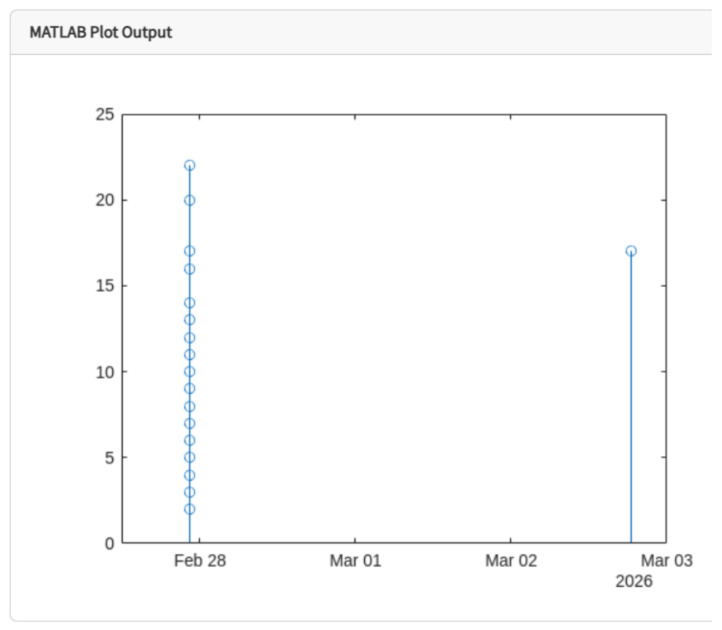
Discrete visualization of compressor vibration obtained through MATLAB processing.

The processing routines implemented in MATLAB also allowed a discrete analysis of the vibration signal in order to observe the evolution of vibration amplitude during compressor operation, as presented in Figure 14.

### 3.4. Statistical and Correlation Analysis of Monitored Variables

To provide a more rigorous evaluation of the compressor operating conditions, a statistical analysis of the monitored variables was conducted using MATLAB tools integrated within the ThingSpeak platform. The analysis considered a dataset covering a continuous monitoring period of 49 days, ensuring a representative characterization of system behavior under real operating conditions.

The evaluated variables include temperature, electric current, and vibration. The statistical analysis focused on descriptive parameters, correlation assessment, and outlier detection to identify possible anomalies or deviations in system performance.

#### 3.4.1. Descriptive Statistical Analysis

The descriptive statistical results obtained from the analyzed dataset are summarized in Table 5.

The results indicate stable operating conditions, with low standard deviation values, particularly for temperature and vibration. The observed variability in electric current is associated with transient conditions such as compressor startup and load fluctuations.

#### 3.4.2. Correlation Analysis

A correlation analysis was performed to evaluate the relationship between the monitored variables. The obtained correlation matrix is presented in Table 6.

The correlation coefficients are close to zero, indicating that no strong linear relationships exist between the monitored variables under normal operating conditions. This behavior suggests that each variable evolves independently, which is consistent with stable compressor operation without coupled fault conditions.

#### 3.4.3. Outlier Detection

Outlier detection was carried out using a Z-score-based method to identify abnormal measurements in the dataset. The results are summarized in Table 7.

The low percentage of detected outliers confirms the stability of the monitoring system and the reliability of the acquired data. The higher number of outliers in the current signal is associated with transient electrical conditions, particularly during compressor startup.

#### 3.4.4. Graphical Analysis

To complement the statistical analysis, graphical representations of the monitored variables were generated using MATLAB. These visualizations provide an intuitive understanding of the temporal behavior, data distribution, and relationships between variables.

##### Time-Series Analysis

The temporal evolution of temperature, electric current, and vibration was analyzed to evaluate the dynamic behavior of the compressor during the monitoring period.

**Figure 15:**
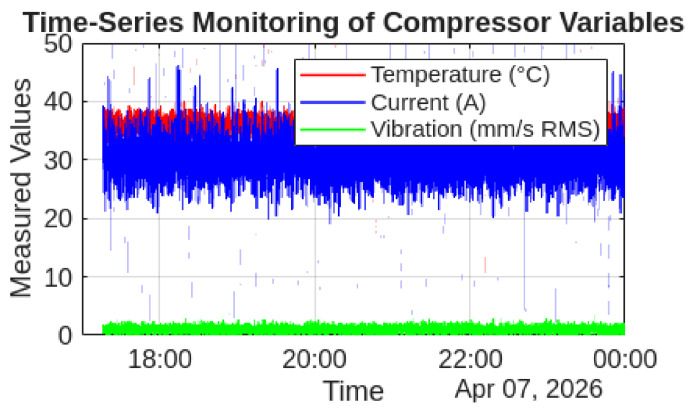
Time-series representation of temperature, electric current, and vibration during the monitoring period.

As observed in Figure 15, the results show stable behavior over time, with no abrupt or sustained deviations that would indicate abnormal operating conditions.

Time-series forecasting using moving average methods is a classical approach for short-term prediction in condition monitoring applications [42,43]. This model was selected for its computational simplicity, interpretability, and suitability for deployment on resource-constrained platforms such as the ThingSpeak MATLAB environment. While more sophisticated approaches—such as ARIMA, exponential smoothing, or LSTM-based neural networks—could provide improved forecasting accuracy [43], their implementation requires larger training datasets, hyperparameter tuning, and greater computational resources than those available in the current architecture. The proposed model is therefore presented as a foundational component of a scalable predictive framework, with advanced models identified as a direct line of future work.

##### Statistical Distribution (Histograms)

**Figure 16:**
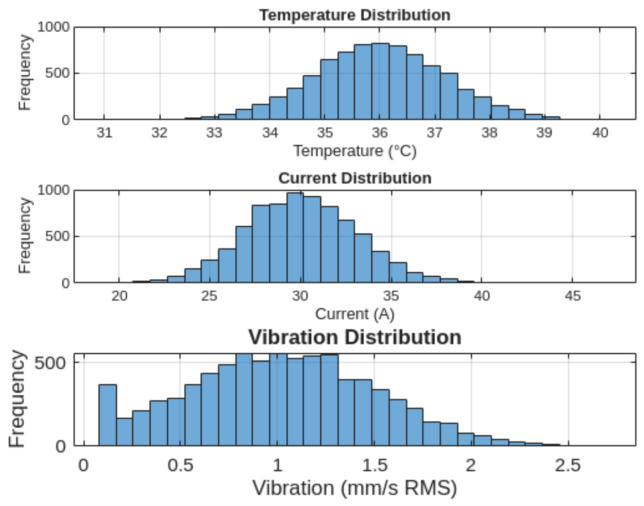
Histogram distributions of temperature, electric current, and vibration.

Histograms were generated to analyze the distribution of each monitored variable and evaluate its variability, as depicted in Figure 16.

The temperature and vibration variables exhibit near-normal distributions, while the current variable shows a wider dispersion due to transient operating conditions.

##### Correlation Analysis (Scatter Plots)

**Figure 17:**
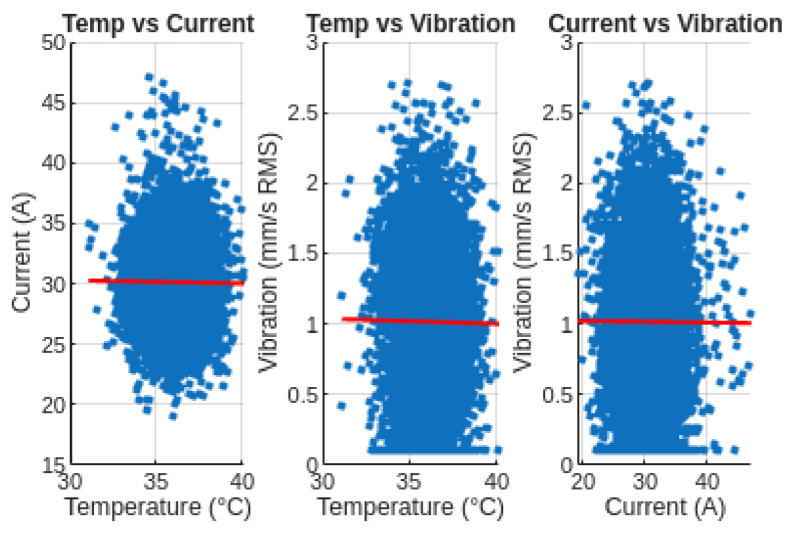
Scatter plot correlation analysis between temperature, current, and vibration. The red lines represent the linear regression fit for each variable pair, illustrating the absence of significant linear correlation.

Scatter plots were used to evaluate possible relationships between the monitored variables (Figure 17).

The absence of clear patterns in the scatter plots confirms the low correlation coefficients obtained in the statistical analysis, indicating independent behavior of the monitored variables.

### 3.5. Anomaly Detection and Predictive Modeling for Maintenance Support

#### 3.5.1. Comparison of Anomaly Detection Methods

To evaluate the contribution of the hybrid approach proposed in this study, a comparative analysis was performed between two anomaly detection strategies applied to the same dataset: a static Z-score threshold method and the proposed hybrid model combining a statistical baseline with moving average prediction error. The comparative results are presented in Table 8.

The hybrid model detects fewer false positives by incorporating temporal context through the moving average predictor, filtering transient fluctuations that the static method flags as anomalies. This behavior is particularly relevant for the electric current signal, where startup transients would otherwise be incorrectly classified as fault indicators by a purely static threshold approach.

To extend the capabilities of the proposed monitoring system beyond traditional condition monitoring, a hybrid anomaly detection and predictive modeling approach was implemented. This approach integrates statistical analysis with time-series forecasting to provide an initial predictive maintenance framework.

Time-series forecasting using moving average methods is a classical approach for short-term prediction in condition monitoring applications [42,43].

The objective of this methodology is to identify abnormal deviations not only based on historical data distribution but also considering the expected future behavior of the monitored variables.

#### 3.5.2. Statistical Baseline Modeling

As a first step, a statistical baseline was established for each monitored variable using historical data. The mean (μ) and standard deviation (σ) were computed, allowing the characterization of normal operating conditions.

Anomaly detection based on statistical deviation was performed using the Z-score:

(1) $$Z=\frac{x-\mu }{\sigma }$$

Values exceeding a threshold of |Z|>3 were classified as anomalies. This method provides a robust mechanism for detecting abrupt deviations in system behavior.

#### 3.5.3. Time-Series Prediction Model

To incorporate predictive capabilities, a time-series forecasting model was implemented using a moving average approach. This model estimates the expected value of each variable based on recent historical observations:

(2) $$\hat{x}\left(t\right)=\frac{1}{N}\sum_{i=1}^{N}x\left(t-i\right)$$

where $\hat{x}\left(t\right)$ represents the predicted value at time *t*, and *N* is the window size.

This approach allows the system to model short-term temporal dynamics and detect deviations from expected behavior.

#### 3.5.4. Prediction Error-Based Anomaly Detection

An additional anomaly detection criterion was defined based on the prediction error:

(3) $$e\left(t\right)=|x\left(t\right)-\hat{x}\left(t\right)|$$

An anomaly is detected when the prediction error exceeds a predefined threshold:

(4) e(t)>k·σ

where *k* is a sensitivity parameter.

This method enables the detection of subtle changes in system behavior that may not be captured by static statistical thresholds.

#### 3.5.5. Implementation in MATLAB and ThingSpeak

The proposed hybrid model was implemented using MATLAB scripts integrated within the ThingSpeak platform. The system performs:

- Real-time acquisition of sensor data
- Statistical baseline computation
- Moving average prediction
- Error-based anomaly detection

This implementation allows near real-time monitoring and predictive analysis of compressor behavior.

#### 3.5.6. Results and Interpretation

The application of the hybrid model to the monitored dataset revealed that the compressor operates within stable conditions, with only minor transient deviations detected.

The prediction model accurately follows the trend of the monitored variables, and the prediction error remains within acceptable limits during normal operation.

Detected anomalies are primarily associated with transient startup conditions, particularly in the electric current signal.

#### 3.5.7. Role of the Hybrid Model in Maintenance Decision Support

The integration of forecasting-based anomaly detection represents a significant step toward data-driven maintenance decision support, as it enables the early identification of deviations from expected system behavior.

Unlike purely reactive monitoring approaches, the proposed model anticipates potential anomalies by comparing real-time measurements with predicted values. These anomaly flags serve as inputs to the condition-based maintenance decision framework presented later in Section 4.

Although the current implementation is based on simple statistical and time-series techniques, the proposed architecture is fully compatible with advanced data-driven models, including machine learning and artificial intelligence approaches such as neural networks, long short-term memory (LSTM) models, and digital twin frameworks. This scalability is a key advantage of the proposed system and enables its evolution toward fully predictive maintenance solutions in future work.

**Figure 18:**
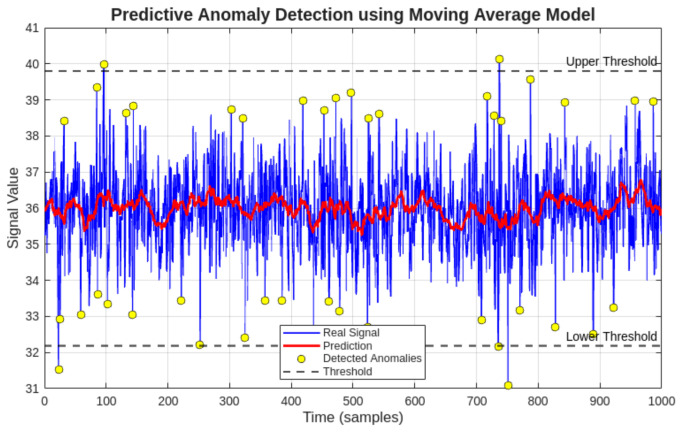
Predictive anomaly detection for compressor temperature using moving average forecasting and error-based thresholding. The results show stable thermal behavior with no persistent anomalies.

**Figure 19:**
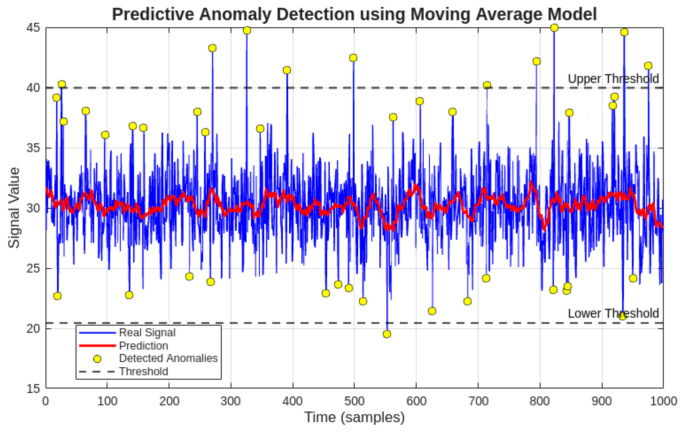
Predictive anomaly detection for compressor electric current. Detected anomalies correspond primarily to transient startup conditions, indicating normal electromechanical behavior.

**Figure 20:**
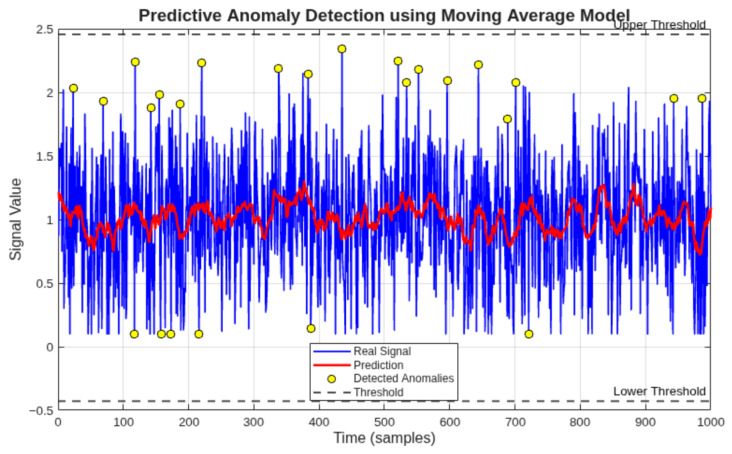
Predictive anomaly detection for vibration signal. The absence of significant anomalies confirms stable mechanical operation within acceptable limits.

The results of the predictive anomaly detection applied to the three monitored variables are presented in Figure 18, Figure 19 and Figure 20. Figure 18 shows the temperature analysis, Figure 19 presents the electric current behavior, and Figure 20 illustrates the vibration signal evolution.

The anomaly detection results for the three monitored variables confirm the stability of the compressor during the analyzed period.

Temperature and vibration signals exhibit minimal deviation from predicted values, indicating steady operating conditions.

In contrast, the electric current signal presents isolated anomalies associated with startup transients, which are expected in normal compressor operation.

These results validate the capability of the proposed predictive model to distinguish between normal transient events and potential abnormal behavior.

### 3.6. Summary of Monitored Variables

To provide an overall view of the compressor operating conditions, the main statistical values of the monitored variables are summarized in Table 9.

## 4. Discussion

The statistical analysis presented in Section 3 provides quantitative evidence of stable compressor operation. The low standard deviation values observed for temperature (1.21 °C) and vibration (0.48 mm/s RMS), together with the reduced percentage of outliers (below 1% for all variables), indicate consistent system behavior under normal operating conditions.

Furthermore, the correlation analysis revealed negligible linear relationships between variables, with correlation coefficients close to zero. This suggests that the monitored variables evolve independently and that no coupled fault mechanisms—such as thermomechanical or electromechanical interactions—were present during the evaluation period. This behavior is characteristic of properly functioning rotating machinery operating under steady-state conditions.

The results obtained from the implemented monitoring system allow the evaluation of the compressor operational behavior through the analysis of three critical variables: temperature, electric current, and vibration. Temperature and current are widely used indicators of thermal and electrical machine health [10], while vibration analysis provides complementary information about mechanical degradation processes in rotating equipment [44]. The combination of these variables constitutes a standard condition monitoring approach in cyber-physical industrial systems [4].

The developed system enabled continuous data acquisition through industrial sensors integrated into a Siemens LOGO! PLC, with information transmitted to a cloud-based analysis platform using Industrial Internet of Things (IIoT) technologies. This architecture facilitates the remote supervision of industrial equipment and allows real-time analysis of operational variables, representing a significant advantage over traditional periodic inspection methods [2,7].

### 4.1. Temperature Variable Analysis

The results obtained for the temperature variable show values ranging between 33 °C and 39 °C during the analyzed operating period. This behavior indicates that the compressor operates within a stable thermal range consistent with the normal operating conditions of similar equipment.

The average temperature recorded, ranging between 34 °C and 38 °C, corresponds to typical values observed in the casing of hermetic compressors during continuous operation. These values suggest that the compressor cooling system operates properly and that no overheating conditions occurred during the monitoring period.

From a condition monitoring perspective, abnormal temperature increases are usually associated with lubrication problems, mechanical overload, or deficiencies in the system’s thermal dissipation. However, the values recorded in this study do not show significant deviations from the expected ranges for normal equipment operation, indicating an adequate thermal behavior.

Additionally, the low variability observed in the temperature signal suggests stable thermal dissipation conditions and consistent compressor load. From a diagnostic perspective, the absence of abrupt temperature fluctuations reduces the likelihood of intermittent faults or lubrication deficiencies during the monitoring period.

### 4.2. Electric Current Variable Analysis

The analysis of the electric current consumed by the compressor shows values ranging between 25 A and 42 A, with a rated current close to 29 A under normal operating conditions. These results fall within the typical operating ranges for electric motors used in industrial compressors.

The maximum recorded value of 42 A mainly corresponds to transient conditions associated with the startup of the electric motor. During startup, it is common for electric motors to exhibit currents significantly higher than the rated current due to the electromechanical characteristics of the acceleration process.

The comparison between the recorded values and the nominal value indicated on the compressor nameplate allows us to conclude that the system operates within acceptable electrical conditions. No sustained current increases were observed that could indicate mechanical overload conditions or failures in the electrical system.

Continuous monitoring of electric current represents an important tool in the diagnosis of machinery driven by electric motors, as it allows the detection of anomalies associated with mechanical wear, misalignment, or faults in the electrical components of the system [45,46].

The moderate variability observed in the current signal, reflected in its higher standard deviation compared to the other variables, is consistent with normal operational dynamics, including startup transients and load variations. The absence of persistent overcurrent conditions suggests that no mechanical binding, electrical faults, or abnormal loading scenarios occurred during the evaluated period.

### 4.3. Vibration Variable Analysis

Vibration analysis is one of the most widely used techniques in the condition monitoring of rotating machinery, as it allows the early detection of mechanical faults such as imbalance, misalignment, bearing wear, or structural problems [25].

In the present study, the recorded vibration values range between 0.10 mm/s and 2.71 mm/s RMS, with an average operating range between 0.8 mm/s and 1.5 mm/s RMS. The majority of the recorded values fall within the acceptable limits established by the ISO 10816-3 standard for industrial rotating machinery.

According to ISO 10816-3 iso [18], vibration velocity values below 1.80 mm/s RMS correspond to Zone A or Zone B, which are associated with acceptable operating conditions for industrial machinery. The majority of the recorded vibration levels in this study fall within these zones, indicating that the compressor operated predominantly under conditions classified as satisfactory for long-term operation.

It is worth noting that, while the average operating range remained within Zone B, a subset of samples concentrated in the final days of the monitoring period reached values up to 2.71 mm/s RMS, placing them in ISO 10816-3 Zone C. This emergent pattern is analyzed in detail in Section 4 (Validation of the CBM Framework on the 49-Day Dataset).

Continuous vibration monitoring allows the detection of progressive changes in the dynamic behavior of machinery, which represents a fundamental tool for the implementation of condition-based and predictive maintenance strategies.

### 4.4. System Behavior Interpretation

Although the monitored variables remained within acceptable ranges, it is important to note that the absence of anomalies in the analyzed dataset does not guarantee fault-free operation under all conditions. The results correspond to a specific operating period and do not include extreme load scenarios or induced fault conditions.

Therefore, the current analysis should be interpreted as a baseline characterization of normal system behavior, which can be used as a reference for future anomaly detection and predictive maintenance strategies.

### 4.5. Proposed Condition-Based Maintenance Framework

Building on the statistical characterization and the hybrid anomaly detection model presented in Section 3, this subsection introduces and validates a condition-based maintenance (CBM) decision framework. The framework is one of the central scientific contributions of this work, as it translates raw sensor data into explicit, standard-referenced maintenance actions.

The framework integrates three reference sources: (i) the ISO 10816-3 vibration severity classification for Class I rotating machinery; (ii) the Copeland ZR144KCE-TF5 manufacturer specifications, in particular the Maximum Continuous Current (MCC = 61.5 A) and nominal thermal operating envelope; and (iii) the empirical 49-day baseline statistics reported in Table 5. Unlike purely data-driven thresholds, this combination anchors the decision logic to industry standards and to equipment-specific limits, improving its transferability to similar compressors.

Based on these sources, six operating conditions and their corresponding maintenance actions are defined in Table 10.

In the present study, the 49-day dataset was processed through this framework to evaluate its behavior under real operating conditions. The obtained results indicate that the analyzed compressor operates within acceptable ranges for the three monitored variables. No critical conditions requiring immediate corrective maintenance were identified within the analyzed dataset; however, this conclusion is limited to the evaluated operating conditions.

Continuous monitoring of these variables allowed the establishment of a baseline reference for the operational behavior of the equipment. This information is fundamental for detecting potential future variations associated with progressive wear of system components [47].

### 4.6. Validation of the Condition-Based Maintenance Framework on the 49-Day Dataset

To move beyond a purely descriptive proposal, the condition-based maintenance framework defined in Table 10 was applied retrospectively to the 49-day monitoring dataset. Each acquired sample was classified into one of the six operating conditions defined by the framework, using the combined state of temperature, current, and vibration at the corresponding time instant. The results are summarized in Table 11.

#### 4.6.1. Sampling Note

The ThingSpeak channel accumulated 1,410,589 raw records during the 49-day acquisition period, corresponding to the 1-s PLC transmission rate described in Section 2. The validation analysis was performed on 8000 samples uniformly retrieved through the ThingSpeak REST API (its maximum single-query limit), which yields a representative resampled series at approximately one sample every 8.8 min. This resolution is consistent with the timescales of the thermal, electrical, and mechanical phenomena monitored in this study and preserves the descriptive statistics reported in Table 5. The full-resolution dataset remains stored on the ThingSpeak platform and is available for further analysis, including higher-frequency spectral studies proposed as future work.

#### 4.6.2. Interpretation of the Classification

The framework classified 93.36% of the resampled dataset as normal operation (Zones A–B), confirming that the compressor operated predominantly within acceptable limits during the evaluation period. The remaining 6.64% distributes across five non-normal conditions, each of which provides specific diagnostic information:

##### Possible Mechanical Wear (Zone C, 5.64%)

A total of 451 samples exhibited vibration levels at or above 1.80 mm/s RMS while temperature and current remained within normal ranges. The mean vibration in this subset was 2.00 mm/s with a peak of 2.71 mm/s, which corresponds to the upper boundary of ISO 10816-3 Zone C for Class I rotating machinery. A cluster analysis of the associated timestamps shows that these events concentrate in the final days of the monitoring period, particularly around 7 April 2026, suggesting a progressive increase in vibration amplitude that the framework would classify as “unsatisfactory” and recommend for mechanical inspection within 3 months. This behavior is consistent with the early stages of mechanical degradation reported in the literature for scroll compressors [22,25].

##### Possible Thermal Issue (Zone B–C, 0.61%)

The 49 samples in this category correspond to isolated temperature excursions above 39 °C without concurrent electrical or mechanical anomalies, consistent with transient load peaks rather than sustained overheating.

##### Possible Electrical Overload (Zone B–C, 0.34%)

The 27 samples classified here correspond to startup transients with current above 42 A, which are also flagged by the hybrid anomaly detection model (Table 8). This cross-validation confirms that the statistical detector and the rule-based CBM framework identify the same electrical events.

##### Anomalous and Critical Conditions (Zone C–D and Zone D, 0.05%)

Four samples in total satisfy a vibration anomaly and a secondary anomaly (thermal or electrical) simultaneously. The single Zone D sample, recorded on 7 April 2026 at 18:15, combined a current of 45.33 A with a vibration of 1.91 mm/s RMS, triggering the immediate-intervention recommendation. The temporal proximity between this event and the cluster of Zone C mechanical-wear samples reinforces the diagnostic value of the framework: an isolated Zone D event preceded by a rising vibration trend constitutes a clearer maintenance signal than either variable considered in isolation.

#### 4.6.3. Framework Validation

These results demonstrate three relevant properties of the proposed framework. First, it does not over-classify normal operation: the 93.36% Zone A–B rate is consistent with the stable statistical profile reported in Table 5 and with the low outlier percentages obtained from the Z-score analysis (Table 7). Second, it detects a genuine emergent condition in the monitored equipment: the concentration of Zone C vibration events in the final days of the monitoring period, together with the single Zone D combined event, suggests an incipient mechanical trend that would not have been visible from any single variable in isolation. Third, the framework maintains consistency with the hybrid anomaly detector on the electrical events, supporting its integration as a decision layer on top of the statistical detector. The detected Zone C/D trend also motivates the extension of the monitoring period and the fault-injection experiments identified as future work in Section 4.7.

In this context, the use of monitoring platforms based on IIoT technologies enables continuous recording of operational information and facilitates the analysis of trends in equipment behavior, contributing to improved reliability of industrial systems and to the optimization of maintenance strategies through real-time operational data [2,9].

### 4.7. Scope and Limitations of the Proposed Framework

The framework proposed in this work corresponds to a condition-based maintenance approach: maintenance actions are triggered by the observed state of the equipment, referenced to industry standards and manufacturer specifications. This should be distinguished from predictive maintenance in its strict sense, which additionally requires a validated prognostic model capable of estimating the remaining useful life (RUL) of the equipment from labeled fault data.

In the present study, the dataset corresponds exclusively to normal operating conditions over a 49-day window. Because no fault events were observed and no controlled degradation experiments were performed, the hybrid anomaly detection model and the CBM framework are validated in terms of internal consistency and sensitivity to transient events, but not yet in terms of fault-detection accuracy against labeled ground truth.

Accordingly, the contribution of this work is positioned as a validated condition-based maintenance framework with an initial predictive component (moving-average forecasting and error-based detection), rather than a fully validated predictive maintenance solution. Extending the framework toward predictive maintenance in the strict sense requires: (i) labeled fault datasets obtained either from long-term monitoring across multiple units or from controlled fault injection experiments; (ii) supervised learning models (e.g., LSTM, gradient boosting, digital twin approaches); and (iii) quantitative evaluation using metrics such as precision, recall, F1-score, and RUL estimation error. These directions are explicitly identified as future work.

### 4.8. Comparison with Existing Monitoring Approaches

Compared to commercial Data Center Infrastructure Management (DCIM) solutions, the proposed architecture offers greater flexibility for integration with legacy industrial equipment through standardized analog interfaces (4–20 mA) and open-source middleware (Node-RED), without dependency on proprietary software ecosystems. While DCIM platforms provide comprehensive facility-level management, they typically require significant licensing costs and vendor-specific hardware integration. The proposed IIoT stack—PLC, Node-RED, and ThingSpeak—represents a cost-effective and technically accessible alternative for monitoring specific critical assets, particularly in contexts where full DCIM deployment is not economically viable. It is acknowledged, however, that the proposed system does not replicate the full management capabilities of commercial DCIM solutions, and its integration within broader facility management frameworks constitutes a relevant direction for future work.

## 5. Conclusions

In this work, a remote monitoring and condition-based maintenance framework based on Industrial Internet of Things (IIoT) technologies was designed, implemented, and experimentally validated for an air-conditioning compressor operating in a real data center environment. The developed architecture integrates industrial sensors, a Siemens LOGO! programmable logic controller (PLC), the Node-RED integration platform, and the ThingSpeak cloud platform, enabling the continuous acquisition, transmission, and analysis of operational data.

The main scientific contributions of this work can be summarized as follows. The first contribution is a field-validated low-cost IIoT architecture, experimentally evaluated on a Copeland ZR144KCE-TF5 scroll compressor over a continuous 49-day monitoring period under real operating conditions. The second contribution is a hybrid anomaly detection model that combines a Z-score statistical baseline with a moving-average prediction-error criterion; the comparative analysis reported in this manuscript shows that the hybrid model reduces false-positive detections with respect to a purely static Z-score baseline, particularly for the electric current signal, where startup transients are filtered out through the temporal context introduced by the moving-average predictor. The third contribution is a condition-based maintenance decision framework that maps the three monitored variables to ISO 10816-3 vibration severity zones and to manufacturer-referenced thermal and electrical thresholds, producing explicit maintenance actions for six operating conditions; the framework was applied retrospectively to the 49-day dataset, classifying 93.36% of the samples as normal operation (Zones A–B) and detecting an emergent mechanical-wear trend (Zone C, 5.64%) concentrated in the final days of the evaluation period, together with one critical combined event (Zone D), thereby validating its diagnostic capability on real operational data. The fourth contribution is a clear scope statement that distinguishes the contribution of this work (a validated condition-based maintenance framework with an initial predictive component) from fully validated predictive maintenance in its strict sense, which requires labeled fault datasets.

Although the Siemens LOGO! PLC used in this implementation belongs to the low-end range of Siemens automation equipment, the proposed architecture is not limited to this device. The modular design of the system–based on standardized analog signal interfaces, Ethernet communication, and open-source integration middleware—allows direct replacement with higher-specification PLCs (e.g., Siemens S7-1200 or S7-1500) without requiring modifications to the Node-RED or ThingSpeak layers. This represents a scalability advantage of the proposed solution for deployments requiring higher processing capacity or additional I/O channels.

During the 49-day evaluation period, the recorded values remained within stable ranges, with temperatures between 33 and 39 °C, electric currents between 25 and 42 A, and vibration levels between 0.10 and 2.71 mm/s RMS. While the overall distribution remained within acceptable limits for the majority of the monitoring period, the condition-based maintenance framework identified a cluster of Zone C vibration events and one Zone D combined event in the final days, demonstrating the framework’s ability to detect emergent conditions that would not be apparent from descriptive statistics alone.

The proposed IIoT architecture demonstrates the feasibility of integrating conventional industrial devices with modern cloud-based data analysis platforms for remote monitoring of critical infrastructures. The use of standardized analog signals (4–20 mA), combined with local PLC processing and data transmission through Node-RED, provides a robust and scalable solution for industrial asset monitoring [9,14].

As future work, the authors propose to extend the monitoring system with labeled fault datasets obtained through controlled fault injection experiments or long-term multi-unit deployments; to evaluate advanced supervised learning models (such as LSTM networks, gradient boosting, and digital twin frameworks) against the proposed hybrid baseline using standard classification metrics (precision, recall, F1-score); to incorporate quantitative estimation of remaining useful life (RUL) in order to evolve the framework toward predictive maintenance in its strict sense; and to assess the generalizability of the proposed architecture across multiple compressor units and different operating environments, including quantitative evaluation of communication latency, packet loss, and sensor calibration against reference instruments.

## Figures and Tables

**Table 1:** Instrumentation used for monitoring compressor variables within the IIoT data acquisition system.

Monitored Variable	Sensor Type/Model	Measurement Range	Output Signal/Interface
Vibration	Piezoelectric RMS velocity transmitter/Hansford HS-420S (Hansford Sensors Ltd., Wellingborough, UK)	0–50 mm/s	Signal converter 4–20 mA to 0–10 V (surface-mounted)
Electrical current	AC current transducer/JXK-14 (Anhui Qidian Automation Technology Co., Ltd., Anhui, China)	0–100 A	0–10 V (installed in electrical panel)
Surface temperature	PT100 RTD probe with integrated transmitter (Cheemi Technology Co., Ltd., Nanjing, China)	0–200 °C	4–20 mA (surface-mounted)

**Table 2:** Temperature values recorded during compressor operation.

Parameter	Value
Maximum temperature	39 °C
Minimum temperature	33 °C
Average operating range	34–38 °C

**Table 3:** Electric current values recorded during compressor operation.

Parameter	Value
Maximum current	42 A
Minimum current	25 A
Rated current	29 A

**Table 4:** Vibration values recorded during compressor operation.

Parameter	Value
Maximum vibration	2.0 mm/s RMS
Minimum vibration	0.3 mm/s RMS
Average vibration range	0.8–1.5 mm/s RMS

**Table 5:** Descriptive statistical analysis of monitored variables over the evaluation period.

Variable	Mean	Std Dev	Min	Max
Temperature (°C)	36.00	1.21	31.08	40.13
Current (A)	30.13	3.23	19.10	47.08
Vibration (mm/s RMS)	1.02	0.48	0.10	2.71

**Table 6:** Correlation matrix between monitored variables.

	Temperature	Current	Vibration
Temperature	1.0000	−0.0099	−0.0094
Current	−0.0099	1.0000	−0.0039
Vibration	−0.0094	−0.0039	1.0000

**Table 7:** Outlier detection results for monitored variables.

Variable	Outliers	Percentage
Temperature	19	0.24%
Current	57	0.71%
Vibration	16	0.20%

**Table 8:** Comparison of anomaly detection methods applied to the monitored dataset.

Method	Temperature Anomalies	Current Anomalies	Vibration Anomalies
Static Z-score (|Z|>3)	19	57	16
Hybrid moving average + error	12	43	9

**Table 9:** Summary of monitored operational variables.

Variable	Minimum	Maximum	Average Range
Temperature (°C)	33	39	34–38
Current (A)	25	42	25–29
Vibration (mm/s RMS)	0.3	2.0	0.8–1.5

**Table 10:** Condition-based maintenance strategy derived from monitored operational variables, with vibration severity zones referenced to ISO 10816-3 and temperature and current thresholds based on the Copeland ZR144KCE-TF5 manufacturer specifications and recorded baseline values.

Detected Operating Condition	Temp. (°C)	Current (A)	Vibration (mm/s RMS)	ISO 10816-3 Zone/Status	Recommended Maintenance Action
Temperature, vibration, and current within normal ranges	≤39	≤42	<1.80	Zones A–B Normal operation	Maintain preventive maintenance program every 9 months
Temperature out of range with vibration and current within normal values	>39	≤42	<1.80	Zones B–C Possible thermal issue	Preventive inspection of the cooling system within 6 months
Current out of range with temperature and vibration within normal conditions	≤39	>42	<1.80	Zones B–C Possible electrical overload	Evaluation of the electrical system and inspection within 1 month
Normal temperature with vibration out of range	≤39	≤42	1.80–2.80	Zone C Possible mechanical wear	Perform technical inspection and verification of mechanical components within 3 months
Temperature out of range or vibration out of range with current within normal values	>39	≤42	>1.80	Zones C–D Anomalous condition	Schedule corrective action within a period shorter than 1 month
Elevated temperature, overload current, or critical vibration (>2.80 mm/s RMS)	>39	>42	>2.80	Zone D Critical condition	Immediate system intervention and technical inspection of the compressor

ISO 10816-3 vibration severity zones (Class I—small machines): Zone A (<0.71 mm/s): new machinery; Zone B (0.71–1.80 mm/s): acceptable for long-term operation; Zone C (1.80–2.80 mm/s): unsatisfactory, corrective action required; Zone D (>2.80 mm/s): severe damage risk. Temperature and current thresholds are referenced to the Copeland ZR144KCE-TF5 nameplate values (MCC = 61.5 A) and the 49-day baseline recorded in this study.

**Table 11:** Classification of the 49-day monitoring dataset according to the proposed condition-based maintenance framework.

Operating Condition	Samples	Percentage (%)	Recommended Action
Normal operation (Zones A–B)	7469	93.36	Maintain 9-month preventive schedule
Possible thermal issue (Zones B–C)	49	0.61	Cooling system inspection (6 months)
Possible electrical overload (Zones B–C)	27	0.34	Electrical system evaluation (1 month)
Possible mechanical wear (Zone C)	451	5.64	Mechanical inspection (3 months)
Anomalous condition (Zones C–D)	3	0.04	Corrective action (<1 month)
Critical condition (Zone D)	1	0.01	Immediate intervention
Total	8000	100.00	

## Data Availability

The data supporting the results of this study are available from the corresponding author upon reasonable request. The datasets generated during the monitoring process were obtained through the implemented IIoT architecture and stored on the ThingSpeak cloud platform, where they were subsequently processed using data analysis tools such as MATLAB.

## Abbreviations

IIoT	Industrial Internet of Things
IoT	Internet of Things
PLC	Programmable Logic Controller
CBM	Condition-Based Maintenance
RMS	Root Mean Square
ADC	Analog-to-Digital Converter
RTD	Resistance Temperature Detector
API	Application Programming Interface
MCC	Maximum Continuous Current
RUL	Remaining Useful Life
LSTM	Long Short-Term Memory
DCIM	Data Center Infrastructure Management
